# Correlation between vitamin D levels in serum and the risk of dental caries in children: a systematic review and meta-analysis

**DOI:** 10.1186/s12903-023-03422-z

**Published:** 2023-10-19

**Authors:** Zizhan Li, Xiao Wei, Zhongjun Shao, Huan Liu, Shizhu Bai

**Affiliations:** 1 College of Basic Medical Sciences, Army Medical University, Chongqing, 400038 China; 2 Department of Stomatology, Xinqiao Hospital, Army Medical University, Chongqing, 400037 China; 3 Department of Epidemiology, Ministry of Education Key Lab of Hazard Assessment and Control in Special Operational Environment, School of Public Health, Air Force Medical University, Xi‘an, 710032 Shaanxi China; 4 State Key Laboratory of Oral & Maxillofacial Reconstruction and Regeneration, National Clinical Research Center for Oral Diseases, Shaanxi Key Laboratory of Stomatology, Digital Center of School of Stomatology, Air Force Medical University, Xi’an, 710032 Shaanxi China

**Keywords:** Dental caries, Vitamin D, Children, Meta-analysis

## Abstract

**Background:**

Vitamin D plays a crucial role in oral health, and its deficiency is associated to significant changes in oral health diseases. We aimed to explore the relationship between levels of 25-hydroxyvitamin D (25(OH) D) and dental caries in children.

**Methods:**

Four electronic databases were searched by two investigators including PubMed, Embase, Web of Science, and Cochrane Library. Dental caries results were presented as either prevalence or based on the index of primary and permanent teeth/surfaces with decaying, missing, and filled areas, while vitamin D levels were determined through laboratory testing. Two researchers independently selected studies, collected information, assessed risk of bias, and evaluated the study quality. Any disagreements were resolved through discussion.

**Results:**

A total of 13 studies were included, comprising 5 cross-sectional studies, 5 cohort studies, 3 case–control studies, all of which had high methodological quality. Our meta-analysis showed that children with vitamin D deficiency had a 22% higher risk of dental caries than those with normal vitamin D levels, with a relative risk (RR) of 1.22 and a 95% confidence interval (CI) of 1.18 to 1. 25. Further subgroup analysis according to the three types of studies showed that the risk of dental caries in children with vitamin D deficiency was higher than that in normal vitamin D level group (cohort studies: 62%; cross-sectional studies, 19%; and case–control studies, 5%). Additionally, according to age, subgroup analysis also showed that the risk of dental caries in children with vitamin D deficiency was higher than that in normal vitamin D level group (permanent teeth studies, 28%; deciduous teeth studies, 68%; and mixed dentition studies 8%).

**Conclusions:**

Levels of 25 (OH) D have been found negatively associated with dental caries in children, indicating that low vitamin D levels may be considered a potential risk factor to this dental disease.

## Background

Dental caries is a progressive destructive disease of the dental hard tissue under the action of many factors dominated by bacteria [1, 2]. This dynamic process begins when the oral defense systems are disrupted by the sugar-driven dysbiosis of the dental plaque microbiota, leading to demineralization of the dental hard tissues [1, 3]. Enamel hypoplasia is a confirmed risk factor for dental caries, which may promote the attachment and colonization of dental caries pathogens on the tooth surface, increasing susceptibility to dental caries and the risk of early dental caries. Remarkably, a shortage of vitamin D during odontogenesis can cause enamel hypoplasia, thereby rendering teeth more vulnerable to caries [4–7].

The most significant form of vitamin D in the bloodstream is 25-hydroxyvitamin D [25-(OH) D], which is produced in the liver and then further hydroxylated into 1,25-dihydroxyvitamin D [1,25-(OH) 2D] in the kidneys [8]. Vitamin D can be obtained from two primary sources: endogenous vitamin D3 produced after skin exposure to ultraviolet B radiation, and exogenous vitamin D3 found in dietary supplements [9, 10]. Despite seasonal variations, up to 90% of the vitamin D in the human body is generated through endogenous metabolism. Vitamin D plays a crucial role in oral health, and a deficiency in this vitamin has been associated to significant changes in tooth, oral, and craniofacial structure, as well as a number of oral health diseases [4, 11]. Especially, 1, 25-(OH) 2D is the key to the absorption of dietary calcium from the intestinal tract and essential in the formation and development of bones and teeth [12, 13].

Recent studies have discovered that nutritional deficiencies are increasingly linked to oral disorders [14]. The lack of vitamin D during deciduous and permanent tooth formation increases the risk of dental caries [15]. Dental caries and periodontitis, which remain the most prevalent oral diseases worldwide, are complex and multifactorial conditions. Vitamin D deficiency (VDD) rickets and its pathophysiological processes are related to these diseases [16]. Vitamin D has various mechanisms that impact dental health beyond bone metabolism. Now, studies have shown that VDD affects tooth development and can lead to fractures and caries in under-mineralized dentition [17]. Additionally, VDD is associated with poor periodontal health and may be involved in the immune mechanism of periodontal infection [18]. Upon its discovery, vitamin D was speculated to quickly stop and prevent tooth cavities. The hypothesized mechanisms of vitamin D's impact on dental health include improved tooth growth, better dentin mineralization, local fluoride impact, changes in saliva, biochemical composition, and vitamin D-controlled caries activity through immunological variables [1, 17]. Some studies have shown that ameloblasts and odontoblasts are the target cells of 1, 25-(OH) 2D. Vitamin D deficiency in the pregnant women can affect the metabolism of fetal ameloblasts and result in dental enamel hypoplasia in young children, making children are more susceptible to dental caries [19].

Although a wide range of published data is available [20, 21], the relationship between vitamin D and caries remains ambiguous. Therefore, the purpose of this study was to evaluate the association between a higher experience of dental caries and lower serum vitamin D levels in children.

## Materials and methods

When conducting our systematic review and reporting findings, we followed the Preferred Reporting Items for Systematic Reviews and Meta-Analyses (PRISMA) [22]. Given that all of the data in this article were obtained from published literature, no informed consent or ethical approval was required. To ensure the reliability of our results, two researchers independently searched for studies, determined eligibility, extracted data, and evaluated study quality. Any disagreements between the two researchers were resolved through discussion and consensus.

### Search strategy

On January 6, 2023, a search was conducted across four electronic databases: PubMed, Embase, Web of Science, and Cochrane Library with no time restrictions. Vocabulary and grammar were modified to match each database's specifications. The search terms included (Vitamin D [MeSH Terms] OR (25(OH)D) OR (25-hydroxyvitamin D) OR [1, 25-(OH) 2D] OR (1,25-dihydroxyvitamin D) OR (VD)) and (Dental Caries [MeSH Terms] OR (Dental Cavity)) OR (Dental Decay) OR (Carious Lesion) OR (Carious Dentin) OR (Dental White Spot)) and (Child [MeSH Terms] OR Children) with no language restrictions. Relevant reference lists were also manually examined for additional potential entries.

### Inclusion criteria

The systematic review included studies that met the following requirements: (1) Cohort, case–control, and cross-sectional studies investigating the epidemiological association between vitamin D level and dental caries in children; (2) Subjects were children under 19 years old, with deciduous teeth under 6 years old, mixed dentition at 6–12 years old, and permanent teeth over 12 years old; (3) The study's exposure factors were vitamin D deficiency (serum 25 (OH) D < 50 nmol/L as deficiency, ≥ 50 nmol/L as normal vitamin D level) [23]; (4) Outcome indicators included caries, missing teeth, number of filled teeth, and dental caries experience; (5) The literature provided relative risk (RR) and its corresponding 95% confidence interval (95%CI), or there was enough data to calculate them; (6) If a study involving the same population was published multiple times, the study with the largest sample size or the most recent publication was selected. The exclusion criteria were as follows: (1) Repetitive literature; (2) Documents with incomplete or unclear analytical data and inconsistent outcome indicators; (3) Poor quality documents with a lack of original data; (4) Case reports, commentaries, expert opinion, and narrative reviews.

### Data extraction

Two reviewers independently reviewed the literature and extracted the relevant data. The reviewers cross-checked their findings, and any discrepancies were resolved through discussion. During the screening process, they assessed the title and abstract before reading the full text, eliminating any obviously irrelevant material. The extracted data, including the first author's surname, publication year, study design, participant demographics, diagnostic criteria for dental caries, method for assessing 25(OH)D levels, prevalence, DMFT or DMFS indices, individual components of either index, Pearson's correlation coefficients (*r*) and odds ratios (OR), and respective confidence intervals (CI), *P*-values, or other summary effect measures, were recorded in standardized Excel files. If there were no data of interest in the published report, the investigators of the original study were contacted via email to request the unpublished data.

### Quality assessment

The New Castle-Ottawa Scale (NOS) was used to rate the caliber of the included cohort and case–control studies [24]. The scale consisted of nine elements, which were grouped into three categories: Selection, Comparability, and Outcome/Exposure. Two reviewers assessed the following NOS components: representativeness of the exposed cohort, selection of the nonexposed cohort (selection bias), ascertainment of exposure, demonstration of outcome, comparability of cohorts (comparability bias), assessment of outcome, follow-up duration, and adequacy of follow-up of cohorts (outcome bias). The study quality was classified as low, fair, or high based on scores of 0–3, 4–6, and 7–9, respectively. Meanwhile, the quality of cross-section studies was assessed according to the 8 criteria of Joanna Briggs Institute (JBI) guidelines [25], and was considered with low risk of bias when the score > 5.

### Statistical analyses

We evaluated heterogeneity between studies using Chi-square statistics and qualified it by I^2^. I^2^ values were used to determine the level of heterogeneity among included studies, with values greater than 50% indicating significant heterogeneity and values of 0% indicating no observable heterogeneity. To combine the results of each article, we normalized the relative risk using random effects models to do analysis and also pooled the adjusted RR in consideration of the multifactorial causes of dental caries in our present study. Funnel plots was applied to assess publication bias. For all statistical analyses, a two-sided *P*-value of < 0.05 was considered significant. We examined data from RCTs that met our inclusion criteria using Stata version 17 (StataCorp, College Station, TX, USA).

## Results

### Search results and study selection

**Fig. 1:**
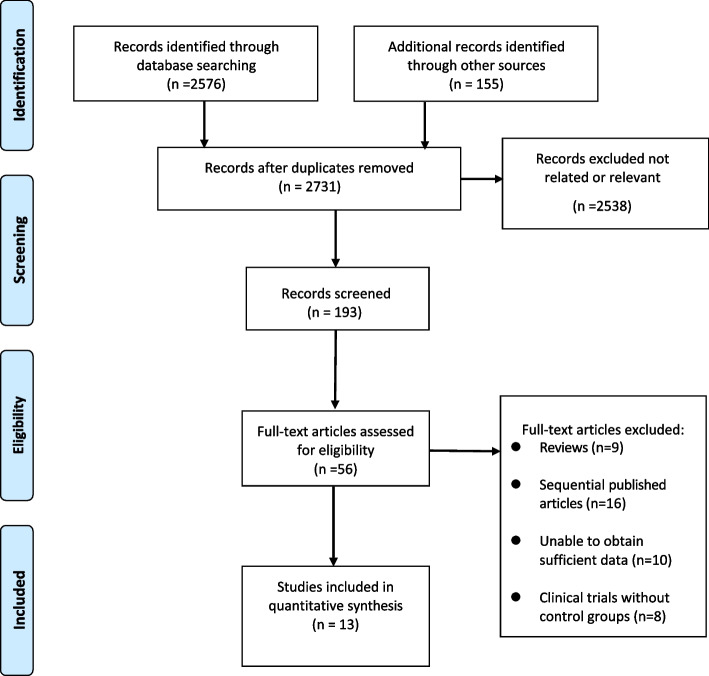
Selection process of included studies

After conducting the initial search of electronic databases, a total of 2731 publications with similar content were retrieved. Through the removal of repetitive literature, examination of titles and abstracts, and strict adherence to inclusion and exclusion criteria, 56 related works were obtained. Subsequently, 43 publications were excluded from further reading, leaving a final set of 13 articles that met the criteria for inclusion [2, 17, 20, 21, 26–34]. Figure 1 illustrates the process and results of the literature screening.

### Study characteristics

The studies included in this systematic review are presented in Table 1. Specifically, the review comprised 13 trials, including three case–control studies [2, 17, 30], five cohort studies [26, 29, 31, 33, 34], and five cross-sectional studies [20, 21, 27, 28, 32]. A total of 19205 patients were included in 13 trials; their mean ages ranged from 1 to 19 years, and the percentage of male patients varied from 47.3% to 75.0%.

**Table 1:** The characteristics and quality assessment of included studies in the meta-analysis

Author	Year	Country	Study design	Study population	Sample size	Site of data collection	Caries diagnosis criteria	Vitamin D deficiency / normality	Score
Carvalho	2021	Portugal	Cohort	< 10 years children	335	the Porto Metropolitan Area (Northern Portugal)	Any Caries: including non-cavitated lesions and/or past treatment	218/117	9
Navarro	2021	Netherlands	Cohort	6 years children	5257	the Rotterdam, Netherlands	Any Caries: including non-cavitated lesions and/or past treatment	2620/2637	8
Akinkugbe	2019	USA	Cross-sectional	12 ~ 19 years adolescents	2579	the National Health and Nutrition Examination Survey, USA	Cavitated Lesions	916/1663	8
Seminario	2018	USA	Cross-sectional	1 ~ 6 years children	276	the Seattle Children's Hospital (SCH), USA	Any Caries: including non-cavitated lesions and/or past treatment	138/138	7
Kim	2018	Korea	Cross-sectional	10 ~ 12 years children	1688	the Korea National Health and Nutrition Examination Survey, Korea	Any Caries: including non-cavitated lesions and/or past treatment	1038/650	7
Gyll	2018	Sweden	Cohort	6 years children	85	Umeå and Malmö, Sweden	Any Caries: including non-cavitated lesions and/or past treatment	50/35	9
Deane	2017	Canada	Case control	6 years children	266	the Misericordia Hospital and community, Canada	Non-Cavitated and Cavitated Lesions	179/87	9
Wagner	2016	Germany	Cohort	3 ~ 4 years children	755	Jena, Germany	Any Caries: including non-cavitated lesions and/or past treatment	60/695	9
Herzog	2016	USA	Cross-sectional	5 ~ 12 years children	1103	the National Health and Nutrition Examination Survey, USA	Non-Cavitated and Cavitated Lesions	343//760	8
Schroth	2016	Canada	Cross-sectional	6 ~ 11 years children	1017	the Canadian Health Measures Survey	Cavitated Lesions	127/890	7
Dudding	2015	Britain	Case control	6 ~ 9 years children	5545	the South West of England	Non-Cavitated and Cavitated Lesions	1952/3593	8
Schroth	2013	Canada	Case control	3 ~ 5 years children	261	the city of Winnipeg, Manitoba, Canada	Non-Cavitated and Cavitated Lesions	43/218	8
Schroth	2012	Canada	Cohort	< 6 years children	38	the southern Manitoba, Canada	Any Caries: including non-cavitated lesions and/or past treatment	32/6	9

*NA* Not available

### Results of quality assessment

In present study, we used the New Castle-Ottawa Scale (NOS) and JBI guidelines to evaluate the included study’s methodological quality. Our analysis revealed that three studies scored 7 points, while five studies received a score of 8 points and five studies achieved a score of 9 points which revealed low risk of bias as shown in Table 1. Additionally, no evidence of funding bias for any of the studies was noteworthy and none of the trials exhibited baseline imbalances, early stopping bias, or inadequate outcome data.

### The results of meta-analysis

**Fig. 2:**
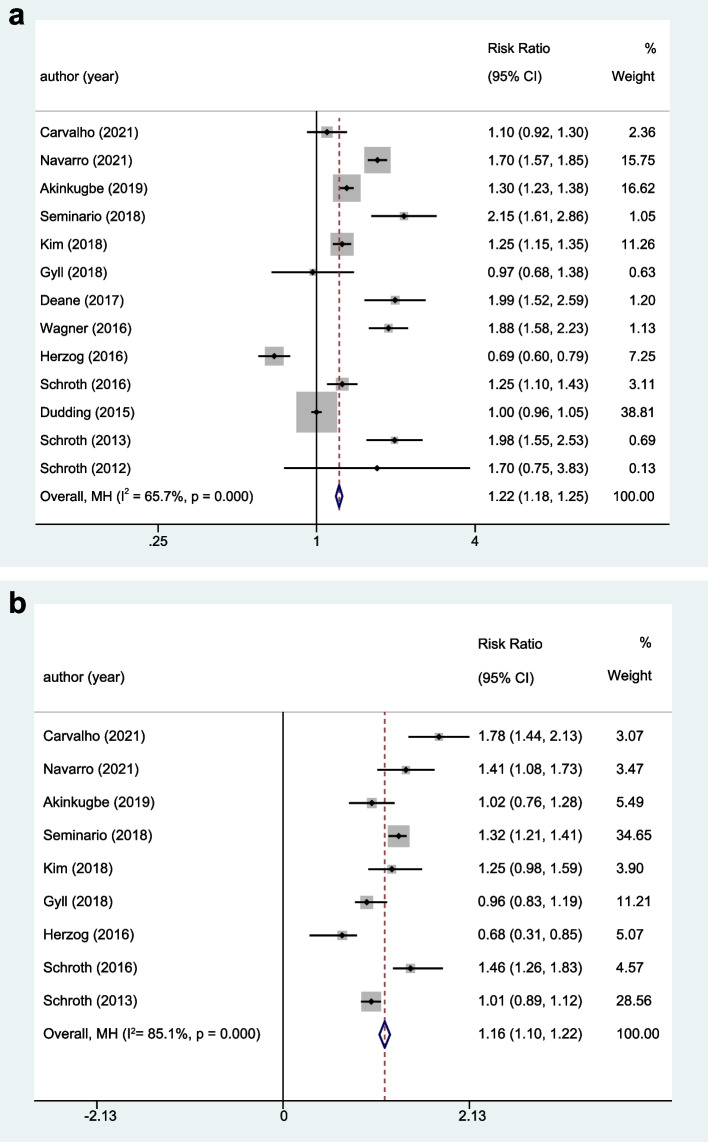
Unadjusted (**a**)/adjusted (**b**) analysis of the correlation between vitamin D level in children and dental caries risk

The unadjusted meta-analysis findings showed significant heterogeneity among the included studies (I^2^ = 65.7%, *P* = 0.000). The random effect model was used, and results showed that patients with vitamin D deficiency had a 22% higher risk of dental caries than those with normal vitamin D levels [unadjusted RR = 1.22, 95% confidence interval (CI): 1.18 ~ 1.25, Fig. 2a]. Considering the multifactorial etiology of dental caries, we conducted an adjusted analysis in our study for confounders which takes some risk factors into account, such as the tooth brushing and socioeconomic factors (adjusted RR = 1.16, 95%CI: 1.10 ~ 1.22, Fig. 2b).

### The results of subgroup analysis

**Fig. 3:**
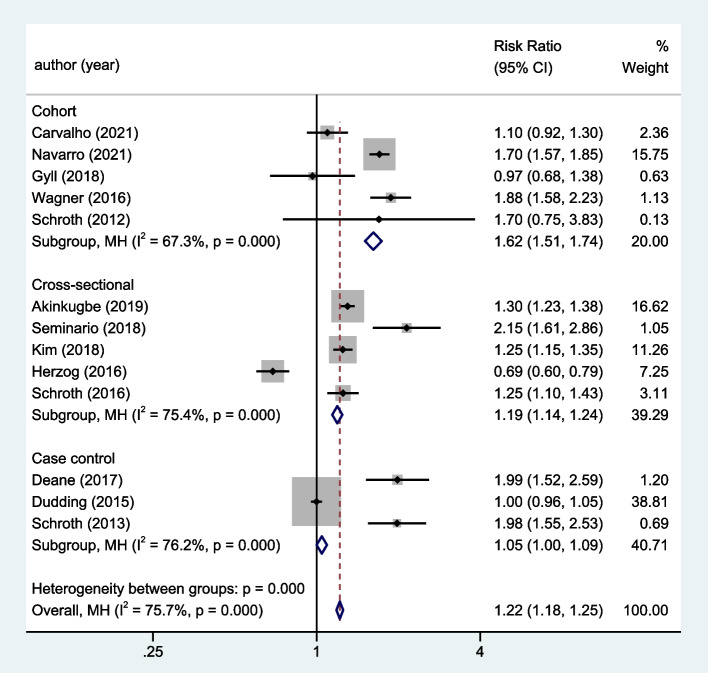
Subgroup analysis according to designed research types for the correlation between vitamin D level and dental caries risk

A total of 13 observational studies were included, including three case–control studies [2, 17, 30], five cohort studies [26, 29, 31, 33, 34], and five cross-sectional studies [20, 21, 27, 28, 32]. The heterogeneity among the five cohort studies, five cross-sectional studies, and three case–control studies were significant (P = 0.000). Our meta-analysis, using the random effect model, found that individuals with vitamin D insufficiency had a 62%, 19%, and 5% greater risk of dental caries than those with normal vitamin D levels in cohort, cross-sectional, and case–control studies, respectively (cohort studies, RR = 1.62, 95%CI: 1.51 ~ 1.74; cross-sectional studies, RR = 1.19, 95%CI: 1.14 ~ 1.24; case–control studies, RR = 1.05, 95%CI: 1.00 ~ 1.09; Fig. 3).

**Fig. 4:**
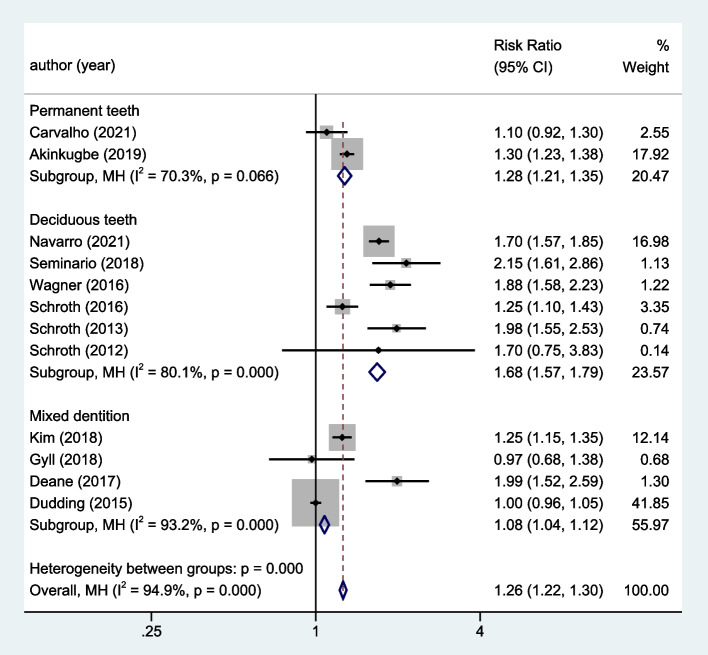
Subgroup analysis according to different age groups for the correlation between vitamin D level and dental caries risk

The subgroup analysis was conducted according to age, including two permanent teeth studies [21, 33], six deciduous teeth studies [17, 20, 26, 28, 29, 34], and four mixed dentition studies [2, 30–32]. The heterogeneity among the two permanent teeth studies, six deciduous teeth studies and four mixed dentition studies were significant (I^2^ = 70.3%, 80.1%, and 93.2%, respectively). Using a random effects model, it was shown that the risk of dental caries was 28%, 68%, and 8% greater in individuals with vitamin D insufficiency than in those with normal vitamin D levels in permanent dentition, deciduous dentition and mixed dentition, respectively (permanent dentition, RR = 1.28, 95%CI: 1.21 ~ 1.35; deciduous dentition, RR = 1.68, 95%CI: 1.57 ~ 1.79; mixed dentition, RR = 1.08, 95%CI: 1.04 ~ 1.12; Fig. 4).

### Publication bias and sensitivity analysis

**Fig. 5:**
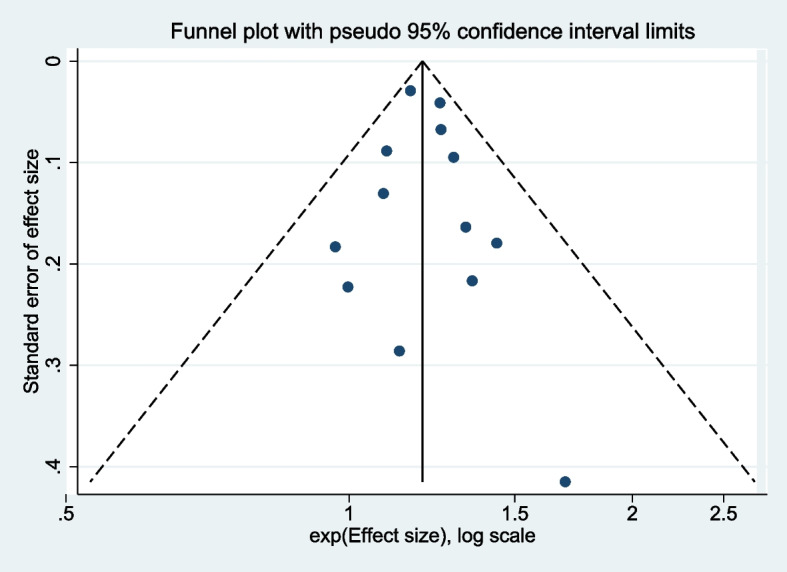
Funnel plot of the correlation between vitamin D level in children and dental caries risk

**Fig. 6:**
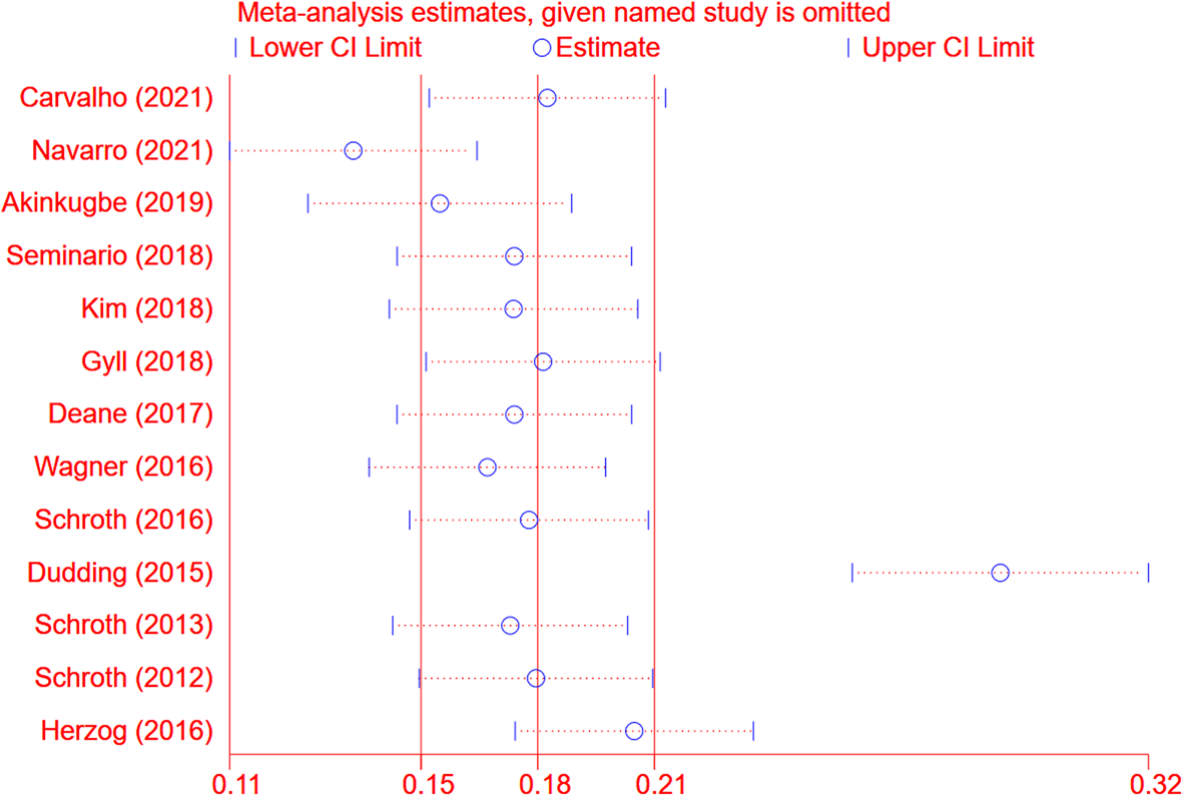
Sensitivity analysis to examine the influence on the summary odds ratio (OR) by omiting individual studies

The funnel plots constructed with the observed study showed symmetry, and no significant publication bias was detected (Fig. 5). Furthermore, to boost the analysis's credibility, sensitivity analysis was performed by excluding each data in turn (Fig. 6). It was demonstrated that the omission of any study did not influence the pooled RRs significantly, indicating the robustness of our results. Remarkably, when we omitted Dudding et al. study, the pooled result led to a certain bias, which contributed the summary RR even large, further confirming our conclusion.

## Discussion

Enamel, dentin, and cementum are the three primary hard tissues that comprise teeth, which are mineralized organs enclosed by alveolar bone. Tooth mineralization occurs in a manner similar to bone, but if mineral metabolism is disrupted, tooth tissue may not mineralize properly [35, 36]. Some studies indicate that vitamin D plays a crucial role in bone and tooth formation and development [37]. Vitamin D facilitates the absorption of calcium and phosphorus in the intestinal tract and their reabsorption in the renal tubules. It also elevates blood levels of calcium and phosphorus, and assists in calcifying the bone matrix through the interaction of parathyroid hormone and calcitonin [38, 39]. Vitamin D insufficiency is linked to a range of systemic illnesses, including cardiovascular disease, metabolic syndrome, malignant tumors, autoimmune disorders, infections, and chronic nephropathy, in addition to bone disorders [40, 41].

The link between vitamin D and dental caries is also a major concern but unclear. One biological viewpoint is that vitamin D works through its receptor, and that LL-37 gene polymorphism may be linked to dental caries [42]. Alternatively, a deficiency in vitamin D may reduce the amount of calcium and phosphate ions in mineralized tissues, resulting in localized tooth decalcification [43]. Other studies suggest that vitamin D regulates immunity and activates the production of antimicrobial proteins, such as defensins and proteases [44], which have multiple antibacterial effects and can effectively inhibit the activity of Gram-negative and Gram-positive bacteria on the tooth surface, increasing susceptibility to dental caries.

At present, it remains ambiguous whether vitamin D levels impact the likelihood of developing dental caries. Doğusal et al. believed serum 25(OH)D concentrations seemed not to have a significant effect on dental caries or molar incisor hypomineralisation in children [45]. Additionally, Herzog et al. found no significant correlation between serum 25(OH)D levels and the incidence of dental caries when the serum 25(OH)D was below 30 nmol/L [27]. Vitamin D was not identified as a predictor of dental caries in a study of Australian twins examining prenatal and developmental risk variables by Silva et al. [46]. However, various trials have shown that vitamin D supplementation can reduce the risk of dental cavities [26, 33, 47]. Kühnisch et al. observed a correlation between an increase in serum 25(OH)D levels and a decrease in dental caries incidence [48]. As blood 25(OH)D levels increase by 10 nmol/L, the incidence of tooth mineralization is reduced by 11%. Hujoel P et al. also claimed that vitamin D exposures in childhood may play a crucial role in caries prevention and revealed vitamin D supplementation could reduce the risk of dental caries by 47% [49]. Additionally, Norrisgaard et al. noted a strong association between vitamin D3 levels and socioeconomic and lifestyle variables, while pregnant women taking vitamin D supplements were less likely to experience enamel abnormalities [50]. These results align with Kühnisch et al.'s findings of a positive correlation between increased blood 25(OH)D concentration at age 10 and a decreased likelihood of enamel abnormalities.

Based on our current meta-analysis, individuals with insufficient levels of vitamin D are 22% more likely to develop dental caries compared to those with normal vitamin D levels. Subgroup analysis showed that children with insufficient levels of vitamin D had a 5% higher risk of dental caries in case–control studies, likely due to the smaller sample size in the case group. Additionally, subgroup analysis based on subject age revealed that the risk of dental caries in children with vitamin D deficiency in deciduous teeth was 68% higher than those with normal levels, possibly due to the lower mineralization of deciduous teeth and young children's inability to maintain proper oral hygiene. Vitamin D's immune regulatory function and antibacterial effects on cariogenic bacteria are more pronounced in this population. The article exhibits some heterogeneity, which may be attributed to various factors. Firstly, different studies used different methods to measure dental caries experience, including prevalence, DMFT/DMFS indices, or subsets of these indices. This lack of uniformity in measurement may lead to inconsistencies in the results. Additionally, the definition of caries varied among studies, with some including only cavitated lesions and others including non-cavitated lesions as well. Furthermore, some studies did not specify whether a cut-off point was used when defining caries with the DMFT index. And our study has several limitations: (1) some of the studies that examined the association between vitamin D levels and dental caries used a cross-sectional design, which can only suggest a correlation and not establish a causative link; (2) there were inconsistencies in how dental caries were measured among the studies, which may make it challenging to interpret the results; (3) the number of studies and patients included in our analysis was small, highlighting the need for larger cohort studies to provide more reliable and accurate data.

## Conclusions

In our study, we demonstrated that dental caries is negatively associated with 25(OH)D levels in youngsters. To improve children's dental health, it is important to ensure sufficient intake of 25 (OH) D during childhood. Healthcare professionals should be mindful that prenatal nutrition and infantile childhood diet can affect the occurrence of dental caries in children.

## Data Availability

The datasets used and/or analyzed during the present study are available from the corresponding author on reasonable request.

## References

[1] Selwitz RH, Ismail AI, Pitts NB (2007). Dental caries. Lancet.

[2] Dudding T, Thomas SJ, Duncan K, Lawlor DA, Timpson NJ (2015). Re-examining the association between vitamin D and childhood caries. PLoS One.

[3] Almoudi MM, Hussein AS, Abu Hassan MI, Schroth RJ (2019). Dental caries and vitamin D status in children in asia. Pediatr Int.

[4] Foster BL, Hujoel PP. Vitamin D in dentoalveolar and rral health//Feldman D. Vitamin D (Fourth Edition). Volume 1: Biochemistry, Physiology and Diagnostics. ScienceDirect: Academic Press. 2018:497–519.

[5] Grant WB (2011). A review of the role of solar ultraviolet-B irradiance and vitamin D in reducing risk of dental caries. Dermatoendocrinol.

[6] Al-Jubori SH, Al-Murad MA, Al-Mashhadane FA (2022). Effect of oral vitamin D3 on dental caries: an in-vivo and in-vitro study. Cureus.

[7] Botelho J, Machado V, Proença L, Delgado AS, Mendes JJ (2020). Vitamin D deficiency and oral health: a comprehensive review. Nutrients.

[8] Hollis BW, Wagner CL, Drezner MK, Binkley NC (2007). Circulating vitamin D3 and 25-hydroxyvitamin D in humans: an important tool to define adequate nutritional vitamin D status. J Steroid Biochem Mol Biol.

[9] Macdonald HM (2013). Contributions of sunlight and diet to vitamin D status. Calcif Tissue Int.

[10] Holick MF (2017). The vitamin D deficiency pandemic: approaches for diagnosis, treatment and prevention. Rev Endocr Metab Disord.

[11] Grant WB (2008). Vitamin D, periodontal disease, tooth loss, and cancer risk. Lancet Oncol.

[12] Bischoff-Ferrari HA, Giovannucci E, Willett WC, Dietrich T, Dawson-Hughes B (2006). Estimation of optimal serum concentrations of 25-hydroxyvitamin D for multiple health outcomes. Am J Clin Nutr.

[13] Holick MF (2006). High prevalence of vitamin D inadequacy and implications for health. Mayo Clin Proc.

[14] Chapple IL, Bouchard P, Cagetti MG, Campus G, Carra MC, Cocco F (2017). Interaction of lifestyle, behaviour or systemic diseases with dental caries and periodontal diseases: consensus report of group 2 of the joint EFP/ORCA workshop on the boundaries between caries and periodontal diseases. J Clin Periodontol.

[15] Wójcik D, Szalewski L, Pietryka-Michałowska E, Borowicz J, Pels E, Beń-Skowronek I (2019). Vitamin D(3) and dental caries in children with growth hormone deficiency. Int J Endocrinol.

[16] Pang X, Yang Z, Wang J, Duan Y, Zhao L, Yu D (2021). Relationship between serum 25OH-vitamin D2 level and vitamin D status of children aged 3–5 years in China. Nutrients.

[17] Schroth RJ, Levi JA, Sellers EA, Friel J, Kliewer E, Moffatt ME (2013). Vitamin D status of children with severe early childhood caries: a case-control study. BMC Pediatr.

[18] White JH (2012). Vitamin D metabolism and signaling in the immune system. Rev Endocr Metab Disord.

[19] Schroth RJ, Lavelle C, Tate R, Bruce S, Billings RJ, Moffatt ME (2014). Prenatal vitamin D and dental caries in infants. Pediatrics.

[20] Seminario AL, Jumani K, Velan E, Scott JM, Latimer J, Schroth RJ (2018). Suboptimal serum vitamin D associated with early childhood caries in special health care needs children. J Dent Child (Chic).

[21] Akinkugbe AA, Moreno O, Brickhouse TH (2019). Serum cotinine, vitamin D exposure levels and dental caries experience in U.S. adolescents. Community Dent Oral Epidemiol.

[22] McInnes MDF, Moher D, Thombs BD, McGrath TA, Bossuyt PM, Clifford T (2018). Preferred reporting items for a systematic review and meta-analysis of diagnostic test accuracy studies: the PRISMA-DTA statement. JAMA.

[23] Ross AC, Taylor CL, Yaktine AL, Del Valle HB (2011). Dietary reference intakes for calcium and vitamin D//Institute of Medicine Committee to Review Dietary Reference Intakes for Vitamin D, Calcium. The National Ccademies Collection: reports funded by National Institutes of Health.

[24] Stang A (2010). Critical evaluation of the Newcastle-Ottawa scale for the assessment of the quality of nonrandomized studies in meta-analyses. Eur J Epidemiol.

[25] Gupta AA, Kheur S, Varadarajan S, Parveen S, Dewan H, Alhazmi YA (2021). Chronic mechanical irritation and oral squamous cell carcinoma: a systematic review and meta-analysis. Bosn J Basic Med Sci.

[26] Schroth RJ, Jeal NS, Kliewer E, Sellers EA (2012). The relationship between vitamin D and severe early childhood caries: a pilot study. Int J Vitam Nutr Res.

[27] Herzog K, Scott JM, Hujoel P, Seminario AL (2016). Association of vitamin D and dental caries in children: findings from the national health and nutrition examination survey, 2005–2006. J Am Dent Assoc.

[28] Schroth RJ, Rabbani R, Loewen G, Moffatt ME (2016). Vitamin D and dental caries in children. J Dent Res.

[29] Wagner Y, Heinrich-Weltzien R (2016). Evaluation of an interdisciplinary preventive programme for early childhood caries: findings of a regional German birth cohort study. Clin Oral Investig.

[30] Rodd C, Deane S, Schroth RJ, Sharma A (2017). Combined deficiencies of 25-hydroxy vitamin D and anemia in preschool children with severe early childhood caries: a case-control study. Paediatr Child Health.

[31] Gyll J, Ridell K, Öhlund I, Karlsland Åkeson P, Johansson I, Lif HP (2018). Vitamin D status and dental caries in healthy swedish children. Nutr J.

[32] Kim IJ, Lee HS, Ju HJ, Na JY, Oh HW (2018). A cross-sectional study on the association between vitamin D levels and caries in the permanent dentition of Korean children. BMC Oral Health.

[33] Carvalho Silva C, Gavinha S, Manso MC, Rodrigues R, Martins S, Guimarães JT (2021). Serum levels of vitamin D and dental caries in 7-year-old children in Porto Metropolitan area. Nutrients.

[34] Navarro CLA, Grgic O, Trajanoska K, van der Tas JT, Rivadeneira F, Wolvius EB (2021). Associations between prenatal, perinatal, and early childhood vitamin D status and risk of dental caries at 6 years. J Nutr.

[35] Foster BL, Nociti FH, Somerman MJ (2014). The rachitic tooth. Endocr Rev.

[36] D'Ortenzio L, Kahlon B, Peacock T, Salahuddin H, Brickley M (2018). The rachitic tooth: refining the use of interglobular dentine in diagnosing vitamin D deficiency. Int J Paleopathol.

[37] Davideau JL, Lezot F, Kato S, Bailleul-Forestier I, Berdal A (2004). Dental alveolar bone defects related to vitamin D and calcium status. J Steroid Biochem Mol Biol.

[38] Svensson D, Nebel D, Nilsson BO (2016). Vitamin D3 modulates the innate immune response through regulation of the hCAP-18/LL-37 gene expression and cytokine production. Inflamm Res.

[39] Reichrath J, Saternus R, Vogt T (2017). Challenge and perspective: The relevance of ultraviolet (UV) radiation and the vitamin D endocrine system (VDES) for psoriasis and other inflammatory skin diseases. Photochem Photobiol Sci.

[40] Skaaby T, Husemoen LL, Pisinger C, Jørgensen T, Thuesen BH, Fenger M (2013). Vitamin D status and incident cardiovascular disease and all-cause mortality: a general population study. Endocrine.

[41] Abhimanyu, Coussens AK. The role of UV radiation and vitamin D in the seasonality and outcomes of infectious disease. Photochem Photobiol Sci. 2017;16(3):314–38. 10.1039/c6pp00355a 28078341

[42] Gombart AF (2009). The vitamin D-antimicrobial peptide pathway and its role in protection against infection. Future Microbiol.

[43] Youssef DA, Miller CW, El-Abbassi AM, Cutchins DC, Cutchins C, Grant WB (2011). Antimicrobial implications of vitamin D. Dermatoendocrinol.

[44] Hewison M (2010). Vitamin D and the immune system: new perspectives on an old theme. Endocrinol Metab Clin North Am.

[45] Doğusal G, Sönmez I, Ünüvar T (2021). Evaluation of serum 25(OH)D levels in obese and normal-weight children with carious and hypomineralized teeth. J Clin Pediatr Dent.

[46] Silva MJ, Kilpatrick NM, Craig JM, Manton DJ, Leong P, Burgner DP (2019). Genetic and early-life environmental influences on dental caries risk: a twin study. Pediatrics.

[47] Chhonkar A, Gupta A, Arya V (2018). Comparison of vitamin D level of children with severe early childhood caries and children with no caries. Int J Clin Pediatr Dent.

[48] Kühnisch J, Thiering E, Kratzsch J, Heinrich-Weltzien R, Hickel R, Heinrich J (2015). Elevated serum 25(OH)-vitamin D levels are negatively correlated with molar-incisor hypomineralization. J Dent Res.

[49] Hujoel PP (2013). Vitamin D and dental caries in controlled clinical trials: systematic review and meta-analysis. Nutr Rev.

[50] Nørrisgaard PE, Haubek D, Kühnisch J, Chawes BL, Stokholm J, Bønnelykke K (2019). Association of high-dose vitamin D supplementation during pregnancy with the risk of enamel defects in offspring: a 6-year follow-up of a randomized clinical trial. JAMA Pediatr.

